# Enabling Expedited Disposition of Emergencies Using Telepsychiatry in Israel: Protocol for a Hybrid Implementation Study

**DOI:** 10.2196/49405

**Published:** 2023-10-17

**Authors:** Ligat Shalev, Moises Bistre, Gadi Lubin, Keren Avirame, Sergey Raskin, Omer Linkovski, Renana Eitan, Adam J Rose

**Affiliations:** 1 School of Public Health Hebrew University Jerusalem Israel; 2 The Jerusalem Mental Health Center Jerusalem Israel; 3 Psychiatric Division Sourasky Medical Center Tel Aviv-Yafo Israel; 4 Department of Forensic Psychiatry Ministry of Health Jerusalem Israel; 5 Department of Psychology Bar Ilan University Ramat Gan Israel; 6 The Gonda Multidisciplinary Brain Research Center Bar Ilan University Ramat Gan Israel

**Keywords:** eHealth, telepsychiatry, digital health service, emergency department, mental health, implementation science, Promoting Action on Research Implementation in Health Services, organizational innovation

## Abstract

**Background:**

Telepsychiatry is the use of virtual communication, such as a video link, to deliver mental health assessment, treatment, and follow-up. Previous studies have shown telepsychiatry to be feasible, accurate compared with in-person practice, and satisfying for psychiatrists and patients. Telepsychiatry has also been associated with reduced waiting times for evaluation and, in some studies, lower admission rates. However, most previous studies focused on using telepsychiatry in community settings and not on involuntary admission.

**Objective:**

The aim of this study is to examine the effectiveness and implementation process of patient assessment for involuntary admissions in the psychiatric emergency department (ED) using a video link.

**Methods:**

This type 1 hybrid implementation study will examine telepsychiatry effectiveness and the implementation process, by comparing telepsychiatry (n=240) with historical controls who had a face-to-face evaluation (n=240) during the previous, usual care period in 5 psychiatric EDs in Israel. A temporary waiver of the standing policy requiring in-person evaluations only, for the purpose of research, was obtained from the Israeli Ministry of Health. During the telepsychiatry phase, clinical staff and patients will join a video call from the ED, while the attending physician will log in elsewhere. The Promoting Action on Research Implementation in Health Services (PARIHS) framework will guide the evaluation of the telepsychiatry implementation process in the ED. PARIHS has the following 3 constructs: (1) *evidence*: staff's opinions regarding the innovation’s viability and practicality, their satisfaction levels with its use, and patients' perceptions of the change; (2) *context*: level of approval of new strategies in the ED, decision-making processes, and the manner in which clinical teams converse and work together; (3) *facilitation*: adequacy of the facilitation efforts using champions reports. Primary clinical outcomes include ED length of stay and violent incidents obtained from medical records.

**Results:**

This study received Helsinki approval from the Ethics Committee of Abarbanel Mental Health Center (174; March 13, 2023), Jerusalem Mental Health Center (22-21; November 6, 2022), Lev-Hasharon Mental Health Medical Center (LH12023; February 12, 2023), Tel-Aviv Medical Center (TLV-22-0656; January 3, 2023), and Sha'ar Menashe (1-4-23; April 18, 2023). Data collection began in July 2023 in 2 study sites and will begin soon at the others.

**Conclusions:**

Telepsychiatry could have significant benefits for patients in the psychiatric ED. Examining telepsychiatry effectiveness in the ED, in addition to identifying the facilitators and barriers of implementing it in different emergency settings, will facilitate better policy decisions regarding its implementation.

**Trial Registration:**

ClinicalTrials.gov NCT05771545; https://clinicaltrials.gov/study/NCT05771545

**International Registered Report Identifier (IRRID):**

DERR1-10.2196/49405

## Introduction

Telepsychiatry is the use of email, chats, telephone, or video link for mental health assessment, treatment, and follow-up [1,2]. In this paper, we focus exclusively on 2-way communication using a real-time video link. There is evidence for the feasibility and effectiveness of telepsychiatry in certain settings, including for children and adolescents [3], adults and older adults [4], postpartum depression [5], developmental disorders like autism [6], and bipolar disorder [7]. Telepsychiatry can be used in different cultures [8] and health settings [9].

Although telepsychiatry is feasible and effective in various situations [1,9], there is less evidence and experience regarding its use for decisions about involuntary admission. Such decisions require the input of an attending psychiatrist, who may currently be required to physically come to the hospital for a face-to-face assessment. Making the patient wait until the attending psychiatrist arrives may increase the risk of additional decompensation, as the patient is already in a vulnerable state. This waiting period and possible decompensation may contribute to violent incidents and worsen the patient’s condition.

In this paper, we discuss the use of telepsychiatry to streamline this process. By allowing an expedited assessment via video link, even when the attending physician is away from the hospital, telepsychiatry can facilitate quicker decisions about involuntary hospitalization. This protocol outlines the evidence supporting a research project to examine this issue empirically and the methods we plan to use.

Although previous studies have discussed the basic conditions to support telepsychiatry in other settings, it does require certain conditions to work well [3]. To conduct a 2-way video conference, one must have adequate internet speed, video and audio quality, and video meeting software [3,10]. It is important to address the security of the video link to prevent malicious intrusions or loss of confidentiality [11]. Both the psychiatrist and patient must be able to operate a computer, join a conference, and operate basic functions such as mute and unmute [3]. Quiet and private rooms must be arranged for both [3,10,11]. Videoconferencing also requires patients to be more active and cooperative than during face-to-face meetings, especially if they are in an unsupervised setting, including home [11,12]. The need for an active and cooperative patient should be considered, especially for patients with cognitive impairment, substance abuse [11], or active psychosis [13]. In our study, a trained staff member, usually a psychiatry resident or a nurse, will be physically present with the patient to support the video chat [3].

Those who wish to use telepsychiatry should be convinced of its accuracy. In fact, a narrative review of 134 articles, mostly from community settings, showed that telepsychiatry can be just as accurate as in-person meetings [9]. However, we are aware of only 2 studies that examined the accuracy of telepsychiatry in emergency settings. In one study from 2014, the authors examined the concordance between clinical impressions via telepsychiatry or in-person evaluation of 73 patients in the psychiatric emergency department (ED) in the United States. A second psychiatrist was present in the room with the patient and completed the assessment independently. In that study, there were no significant differences between raters regarding the recommended disposition, strength or confidence of the disposition recommendation, provisional diagnosis, or the rating of dangerousness [14]. In 2022, our research group published a study of 38 Israeli patients who were referred to the ED for possible involuntary admission. All patients were evaluated both face to face and by video link by 2 different clinicians. The main finding was that there was an extremely high degree of concordance between impressions arrived at under the 2 conditions, especially regarding the need for involuntary admission [15].

In addition to being convinced that telepsychiatry is accurate, one also must be convinced that it is feasible. The question of feasibility can be divided into whether it is feasible for patients as well as for health care providers. For patients, previous work has shown that telepsychiatry is acceptable and highly satisfying in community-based settings [2,9] and in the ED [15-17]. Regarding health care providers, the evidence for feasibility is also generally positive, although more limited. In one study, health care providers had mixed feelings about telepsychiatry before using it [18]. In other studies, after using telepsychiatry, they perceived it to be acceptable [1,17].

In addition to this generally positive impression by clinicians, telepsychiatry has advantages for the health care system. Several studies have documented positive system effects. For example, the use of telepsychiatry contributed to decreased waiting times for patients awaiting psychiatric input in clinical setting (EDs and community clinics), reducing workloads [9,19], and reducing medical costs [20].

In addition to using telepsychiatry in the ED, some studies have examined professionals' impressions of the process of implementing telepsychiatry in the ED. Saurman et al [21] evaluated the perceptions of hospital staff regarding a new telepsychiatry program at a rural ED in Australia. The staff reported that they were satisfied with the program, felt confident in managing patients' cases, and valued the accessibility to mental health experts, which they otherwise would not have in such a remote location. An additional study examined the views of 17 staff members from Canadian EDs, and participants reported that telepsychiatry can improve patient experience and expedite service but can also introduce challenges for an already overloaded ED [22]. According to a 2009 report for the California Health Care Foundation, providers and patients who participated in ED telepsychiatry programs reported high satisfaction from the video meetings and stated that, in their opinion, patient outcomes, such as hospital admission or length of stay, were improved using this method [16].

One study that is worth noting was not conducted in an ED setting but does support the use of telepsychiatry for psychiatric emergencies. A university hospital in Norway developed new telepsychiatry services for emergencies in rural areas that lack psychiatric service availability. The findings showed that having access to experts through telepsychiatry was beneficial for patient involvement, reduced nurses' uncertainty, and served as a safety net for the health care team [23].

Unfortunately, previous studies regarding the feasibility of using telepsychiatry in an ED setting have limitations. These studies examined the practical implementation of telepsychiatry in relatively few settings and with relatively small samples and only characterized a relatively limited range of process outcomes. Additional information about the feasibility of using telepsychiatry in ED settings and the outcomes of patients managed using telepsychiatry would help support decisions by policymakers to adopt this strategy.

Although the previous literature has important gaps, we do already know that there are some barriers to the increased use of telepsychiatry. For example, Cowan et al [19] found that health care providers noted technological challenges, the need for guidance and training on how to use this method, and concerns regarding patients' privacy and safety. Other studies documented the unequal degree of comfort with the use of telepsychiatry by patients, with some patients adapting to the new technology relatively easily and others having more difficulty [24]. Last, several studies documented legal or procedural challenges with using telepsychiatry [3,12].

In some cases, restrictive laws may make it difficult or impossible to use telepsychiatry. In Israel, for example, it is the law that, before they can be hospitalized involuntarily, patients must be evaluated in person by a senior psychiatrist [25]. It is worth noting that such policies were created before it was possible to evaluate patients by video link. Although policy is difficult to modify, a number of jurisdictions around the world found ways to change these rules during COVID-19 lockdowns. This led to a rapid expansion of the use of many kinds of telemedicine, including telepsychiatry [26,27].

Although most previous efforts to use telepsychiatry have been in the relatively low-stress setting of delivering outpatient care, the use of telepsychiatry for ED triage could have great benefits, at least theoretically. Patients with acute psychiatric decompensation may pose a danger to themselves and those around them [28]. Their vulnerable state is complex, as they may be agitated and have poor reality testing [28]. Decompensation is more likely to happen when psychiatric patients are staying in settings that are not well-suited to their needs, such as the ED [29]. The ED is a relatively uncomfortable environment for any patient but is even more stressful for patients with psychiatric emergencies [28]. This can result, ironically, in the patient’s condition worsening, violent incidents directed toward medical staff, and starting the hospitalization with precisely the reverse of the intended therapeutic benefit [29]. To the extent that incorporating telepsychiatry could help move such patients out of the ED faster, this could decrease the strain on the patients, bringing them to a quiet and controlled environment sooner, where they can begin to heal. Thus, introducing telepsychiatry in this setting, although possibly challenging, could also have great benefits.

Although the idea of using telepsychiatry in triage decisions is not entirely novel, relatively few studies on patients' outcomes have been published in this setting. A national survey in the United States in 2016 examined the extent to which telepsychiatry is already in use in some ED settings. The study found that 20% of the EDs used telepsychiatry, with the most common use being to decide which patients to admit and which to send home [30]. Additional studies examined patient outcomes with telepsychiatry in the ED. Narasimhan et al [31] examined 7261 patients who arrived at the ED and underwent a real-time video visit with a remote psychiatrist. Compared with a matched control group, the telepsychiatry group had significantly more outpatient follow-up care and fewer hospitalizations. This, in turn, was associated with a significant reduction in hospital costs. An additional study from the United States examined the use of telepsychiatry in the pediatric ED. The findings showed a significant reduction in patients' length of stay for nonhospitalized patients (ie, for those sent home from the ED) [32].

Previous studies described the use of telepsychiatry in an ED setting but did not specifically examine its use for the purpose of deciding which patients require involuntary admission. In fact, only 1 observational study examined involuntary admissions to the ED via telepsychiatry [33], but this study did not directly compare patient outcomes between telepsychiatry and face-to-face approaches.

To broaden our understanding of successful telepsychiatry implementation in the ED, further studies guided by implementation science frameworks are needed. One example of an implementation science framework is the Promoting Action on Research Implementation in Health Services (PARIHS) framework [34]. PARIHS posits that successful implementation (SI) is a function of 3 inputs: *evidence*, *context*, and *facilitation* [35]. SI is the extent to which the innovation is completely implemented and adopted as part of standard practice, as opposed to being incompletely adopted or resisted. *Evidence* refers to end users’ assessments of the strength of the evidence for the innovation, including their expectations that it will be feasible to use in their setting. *Context* refers to the factors in the environment that support (or resist) the implementation of changes in practice. *Facilitation* refers to the efforts of the research team or champions within the clinical team to promote the change.

Our study will examine the implementation of telepsychiatry in Israeli EDs for involuntary admissions using the PARIHS framework, with attention to characterizing which aspects of *evidence*, *context*, and *facilitation* contributed to SI, or the lack thereof. We will also examine the impact of telepsychiatry on patient outcomes and health care utilization, compared with historical controls managed without telepsychiatry.

## Methods

### Ethics Approval

Our study is being performed in accordance with all relevant guidelines and regulations. Helsinki approval was obtained from the Ethics Committees of Abarbanel Mental Health Center (174; March 13, 2023), Jerusalem Mental Health Center (22-21; November 6, 2022), Lev-Hasharon Mental Health Medical Center (LH12023; February 12, 2023), Tel-Aviv Medical Center (TLV-22-0656; January 3, 2023), and Sha'ar Menashe (1-4-23; April 18, 2023). Any changes in the study protocol will be promptly communicated to the ethic committees, and if necessary, approval for the change will be obtained. Patients will provide verbal assent for telepsychiatry evaluation.

Since the care provided during the study will be the same standard of care for all patients, at least for the duration of the study, it was not deemed necessary to have a data safety monitoring committee. The most easily foreseeable adverse consequences of the change in practice are being measured by the study, and the duration of the intervention period (4 months) is brief. In addition, study data are being collected via chart review and not necessarily contemporaneously within the study period itself. There is not sufficient time during this brief study period to conduct interim analyses. Following an analysis of the study outcomes, a policy decision will be made on the part of Israel’s Ministry of Health whether to continue or end the change in practice. There are no formal plans to compensate patients for any harms that may occur during the study, which are likely to be similar to whatever harms could occur under usual care.

### Study Design

We will implement a multisite study in 5 Israeli EDs to evaluate the use of telepsychiatry compared with the regular face-to-face method. Under the Mental Health Act regarding involuntary hospitalizations in Israel, all involuntary hospitalizations require an in-person evaluation by the attending psychiatrist [25]. Our study received a limited waiver of this policy for the purposes of research, after which the results of our study will be used to re-evaluate whether the current policy should be changed.

In our study, there will be clinicians, including psychiatry residents and nurses, physically present. However, using the innovation, the attending physician will evaluate the patient via video link, with the conversation facilitated by another provider who is physically present. This has numerous potential advantages, in that the patients may enter a calmer environment (ie, the psychiatry ward) or be sent home sooner if they are not admitted. Less time spent in the ED has the potential to reduce violent incidents and improve the patient’s hospital course, by minimizing exposure to a noxious stimulus.

Our study will be a type 1 hybrid implementation study. Hybrid implementation studies aim to shorten the process of translating clinical innovations into practice by simultaneously collecting data about effectiveness as well as the implementation process [36]. A type I hybrid study emphasizes the collection of effectiveness data, often because there is limited previous evidence of effectiveness. However, the team also collects information regarding the contextual factors that supported or impeded implementation, with an eye toward improving the future implementation of this strategy.

Our study will compare the innovation with historical controls. During the pre-intervention period, we will collect data about patient outcomes during a period of 4 months when attending physicians continue to evaluate patients in person, as they have been doing (usual care). During the intervention period, attending physicians will evaluate the patients and make decisions based on a video link. Seasonality would be one possible threat to study validity [37]. To address this, we will collect data using the same 4 months of the year, both in the pre-intervention and intervention periods.

Evaluation of process implementation will be guided by the PARIHS framework [34]. We will characterize the extent to which staff members believe that the innovation will be effective and will be feasible to use in their context and how this belief contributes to SI [34]. We will characterize how the context, including relationships among staff, styles of administrative leadership, and organizational attitudes toward change and quality improvement, at each of our 5 medical centers contributes to or detracts from SI. Finally, we will characterize how the efforts of champions within the clinical staff, known as internal facilitators, did or did not help promote SI [35].

### Study Settings

The study will be conducted in 5 psychiatric EDs in Israel. One of the study sites is a general hospital, so psychiatric emergency patients are evaluated and treated in a special section of the general ED. The other 3 hospitals are dedicated psychiatry hospitals, meaning that the ED is only used for psychiatric emergencies. All sites are located in the densely populated center of Israel, in or near Tel Aviv and Jerusalem, where most of the country’s population lives [38]. All 5 hospitals are moderate to large in size.

### Participants

This study will include 2 population groups: patients and hospital staff.

#### Patients

We anticipate that our 5 hospitals will collectively evaluate at least 60 patients per month for involuntary admission. Given that each hospital will have a 4-month pre-intervention period and a 4-month intervention period, we plan to enroll at least 240 patients in each study period. To be included, patients must be 18 years or older and arrive during hours when the attending physician is not at the hospital (evening and night shifts). Due to the waiver we received for this study and the limitations that were placed on the waiver, we will exclude patients who are brought for evaluation by police after being detained or charged with a crime. Only patients needing an attending assessment under the Mental Health Act regarding involuntary hospitalizations will be included. Patients will provide verbal assent to be evaluated by video link; patients who do not agree will be evaluated in person. Although we will endeavor to support patients who have difficulty participating in a video link, there may be some patients who are unable to communicate adequately by video link, and we will exclude them from the study. To maximize study generalizability, no other exclusion criteria exist.

With an anticipated 240 patients in the control arm and 240 patients in the intervention arm (4 X 60), we will have statistical power to show a relatively small effect size for most outcomes. For example, we will be able to show a difference of as little as 15 minutes of ED time.

#### Hospital Staff

We will collect data about implementation from all those who are involved with administrating, supervising, or staffing the psychiatric ED. Key staff will include the hospital director, department director, unit director, service manager, senior physicians, psychiatry residents, and nursing staff.

### Clinical Outcomes

Clinical outcomes are explained in detail in Table 1. Data will be collected in the same manner from the medical record both before and during the intervention. Baseline patient variables will include sex, age, marital status, city of residence, ethnic group (Jewish or Arab), and known psychiatric diagnoses. We will also collect information about the patient’s arrival at the ED, including mode of arrival, legal status, violent incidents pre-arrival, and chronic medications. The primary outcomes for the ED stay, for patients who are admitted involuntarily and for those discharged home, will include time in the ED and violent incidents. Secondary outcomes will include the psychiatric diagnosis given by ED staff, psychiatric status (eg, judgment intact or not intact), and legal status upon release. For patients admitted to hospital, we will also collect information on hospitalizations reversed within 72 hours, which implies that the hospitalization was not necessary, and length of hospital stay. For patients discharged home, we will collect information on those admitted within 7 days, which implies that the patient should have been hospitalized.

**Table 1:** Planned collection of outcome variables for a 6-month pilot study comparing telepsychiatry with usual care (historical outcomes during the pre-intervention period) in the emergency department (ED) setting, for assessment of the need for involuntary admission.

Variable				Data collection method
Background characteristics				Patient sex, age, family status, residence, ethnic group, known psychiatric diagnosis; as well as, for both ED arrival and hospitalization: involuntary arrival or by police or emergency services, legal status, violence incidents pre-arrival, and prescribed medications and dosages
**Primary outcomes**				
	ED length of stay			Measured from the time of arrival in the ED until the patient physically leaves the ED to go home or for hospitalization
	Violent incidents in the ED			As recorded by ED staff and adjudicated by blinded review of the chart by a senior psychiatrist; violent incidents defined as one of the following: (1) degree of restlessness: calm, moderate restlessness, severe agitation, or catatonia; (2) violence type: verbal violence, violence toward property, violence toward others (eg, escort, staff member, other patients)
	Duration of hospitalization (hours)			From the time of ED release until the time of leaving the hospital
	Violent incidents in the ward			As recorded by the hospital staff and adjudicated by blinded review of the chart by a senior psychiatrist
**Secondary outcomes**				
	Psychiatric diagnosis in the ED and hospitalization in case of admission			Psychiatric diagnosis categorized as one of the following: (1) neurodevelopmental disorders, (2) schizophrenia spectrum and other psychotic disorders, (3) bipolar and related disorders, (4) depressive disorders, (5) anxiety disorders, (6) obsessive-compulsive and related disorders, (7) trauma and stressor-related disorders, (8) somatic symptom and related disorders, (9) substance-related and addictive disorders, (10) neurocognitive disorders, (11) personality disorders, (12) adjustment disorder, (13) diagnosis deferred
	Psychiatric status in the ED and hospitalization in case of admission			Psychiatric status to include the presence or absence of the following: (1) disturbed thinking, (2) disturbed perception, (3) disturbed judgement, (4) dangerousness
	Release from the ED and hospitalization in case of admission			Release from the ED and hospitalization in case of admission to include one of the following: (1) discharge to home, (2) discharge to the institution from which the patient came, (3) hospitalization in a general hospital, (4) hospitalization in another psychiatric hospital, (5) police, (6) runaway, (7) other place
	Legal status upon release from the ED and hospitalization in case of admission			Legal status upon release to include one of the following: (1) with consent, (2) compulsory follow-up visits in a community clinic, (3) involuntary hospitalization order, (4) other
	Reversal of admission			Unnecessary admission: involuntary admission that is reversed within 72 hours; measurement of the proportion of admissions reversed within 72 hours
	Admission within 1 week of ED discharge			A patient sent home from the ED without being admitted, who is then admitted within 1 week and should have been admitted the first time
**Implementation process outcomes**				
	**Evidence construct**			
		SHEMESH^a^ questionnaire	A validated tool to evaluate organizational change and identify its barriers; evaluation of the evidence construct achieved via 4 online questions administered to the medical and nursing staff about the strength and feasibility of psychiatric assessment via video link at the ED and how preferable it is compared with the face-to-face method; items rated from 1 (strong disagreement) to 5 (strong agreement)	
		Patient satisfaction with ED course	As they are leaving the ED, patients asked to rate their overall satisfaction with 4 items (overall ED experience, interactions with nurses, interactions with residents, interactions with the attending physician), rated using a visual analog scale from 1 (very dissatisfied) to 5 (very satisfied)	
		Staff satisfaction with the ED work	Medical and nursing staff asked to rate their overall satisfaction with their work in the ED on a scale from 1 (very dissatisfied) to 5 (very satisfied)	
		Medical staff confidence with evaluation	Medical staff asked to rate their overall confidence with evaluating patients sufficiently well to make appropriate decisions about whether they need to be admitted on a scale from 1 (very uncertain) to 5 (very confident)	
	**Context construct**			
		SHEMESH	Evaluation of the context construct achieved via 6 online questions administered to the medical and nursing staff about the acceptability of initiatives, the way decisions are made, and the way medical and nursing teams are communicating and collaborating in the ED; items rated from 1 (strong disagreement) to 5 (strong agreement)	
	**Measurement of successful implementation**			
		Staff's impression of the implementation process	Measured via an open-ended questionnaire to all staff members at the conclusion of the intervention period, to gather their thoughts on the advantages and the challenges of using telepsychiatry; its fit for a variety of patient groups; and in their opinion, the necessary conditions for the use of telepsychiatry	
		Feedback from clinical champions	Solicitation of ongoing feedback from clinical champions located within each site who are responsible for promoting the intervention; feedback to be recorded in a research diary by the researchers and include their impression of success and failure of the implementation process, what would help improve it, and how fully the innovation is being adopted	

^a^SHEMESH: acronym of “SHE'elon Muchanut Ergunit le'SHinuy” in Hebrew, translated from the English name of “Organizational Readiness to Change Assessment.”

### Implementation Outcomes

Evaluation of the implementation process of telepsychiatry will be guided by the PARIHS model. We will collect information on SI and the 3 factors that contribute to it: *evidence*, *context*, and *facilitation*. SI will be assessed according to feedback from site champions at each of the 5 sites, who will share impressions with our team about what is going well or less well in the implementation effort. This feedback will also be used to support tailoring of the intervention across each of the 5 sites. SI will also be investigated by open-ended questionnaires administered to all staff members to understand providers’ impressions of which parts of the intervention succeeded and which should be modified.

The *evidence* construct includes staff perceptions of the strength and feasibility of psychiatric assessment via video link at the ED, how preferable it is compared with the face-to-face method, and psychiatrist confidence in the accuracy of admission decisions made using video link. The *context* construct includes the acceptability of initiatives in the ED, the way decisions are made, and the way medical and nursing teams are communicating and collaborating in the ED. Information on the *facilitation* construct and the adequacy of our facilitation efforts will come mostly from the site champions.

### Data Collection

Data collection will include patient outcomes and implementation outcomes. Patient outcomes will be collected through hospital medical records using trained assessors. Process outcomes will be collected using questionnaires. The Organizational Readiness to Change Assessment survey is a validated tool created to evaluate organizational change and innovation and identify the barriers through the prism of the PARHIS framework [35]. For the purpose of this study and initially for the use of others, our group developed and validated the Hebrew version of this questionnaire, named “SHE'elon Muchanut Ergunit le'SHinuy” (English: Organizational Readiness to Change Assessment) and abbreviated as SHEMESH. The SHEMESH will examine medical and nursing staff perceptions about the evidence of telepsychiatry and its implementation in the ED context. The SHEMESH has high Cronbach alpha scores for the 2 scales (total of 10 items on a 5-point Likert scale): *evidence* (4 items): α=.887; *context* (6 items): α=.852 [39]. The questionnaire will be administered online once before implementing telepsychiatry and again 3 months after the implementation. Staff will be asked to rate their overall agreement with statements on a scale from 1 (strong disagreement) to 5 (strong agreement).

Data about the *evidence* construct will be also collected through the following questionnaires: patient satisfaction questionnaire, staff satisfaction survey, and medical staff confidence in psychiatric evaluations survey.

The patient satisfaction questionnaire will be used to compare the patient experience under usual care with that under the intervention condition. ED patients will be asked 3 questions about their overall experience at the ED and their interactions with medical and nursing staff. Patients will be asked to rate the questions using a visual analog scale from 1 (very bad) to 5 (very good). During the telepsychiatry phase, they will also be asked about their satisfaction with the video link.

The staff satisfaction survey will be administered online for all ED medical and nursing staff, once before implementing telepsychiatry and 3 months after the implementation. Staff will be asked to rate their overall satisfaction with their work in the ED from 1 (very dissatisfied) to 5 (very satisfied)

The medical staff confidence in psychiatric evaluations survey will be administered online before the telepsychiatry implementation and 3 months after the implementation. Staff will be asked to rate their overall confidence in patients' evaluations from 1 (very uncertain) to 5 (very confident) for admission decisions.

Data regarding the SI outcome will be collected using an open-ended questionnaire administered to all staff members at the end of the intervention phase. Staff will be asked about the advantages of and challenges with using telepsychiatry in the ED, its fit for patients from different backgrounds, and their opinion of the necessary conditions for the use of telepsychiatry.

All data collected during the research will be anonymized, entered, and stored in password-protected files on a researcher's (LS) computer.

### Data Analysis and Dissemination of Results

All data will be analyzed using SPSS (version 27.0). Baseline characteristics will be compared between the intervention and historical controls to ensure that the 2 groups are balanced. All normally distributed data will be analyzed using *t* tests or ANOVAs. Data found to be non-normally distributed will be analyzed using Mann-Whitney *U* tests or Kruskal-Wallis *H* tests. Comparisons of percentages between different groups will be analyzed using chi-square tests. Close-ended questions using Likert scale responses will be grouped as negative (1-2), neutral (3), or positive (4-5).

The results of the study will be disseminated to the public via published manuscripts in the peer-reviewed literature, and we also plan to brief the Israeli Ministry of Health on our study findings. Authorship will include all study investigators, including, at a minimum, all site principal investigators and likely others who participated in collecting data. Manuscripts and reports will be written by study investigators, and we will not rely on medical writers. The study protocol is being published with this manuscript, and we intend to share the statistical code with those who request it. Data from the study cannot be made available due to the terms of our study approvals by the relevant research ethics committees of the hospitals involved.

## Results

This study has been funded by the Israel National Institute for Health Policy Research (grant number 2021/77). Data collection began in July 2023 in 2 study sites and will begin soon at the others. Data collection for the pre-period will be performed from the previous year, to match the months during which the pilot program was implemented at that site.

## Discussion

### Overview

We propose an innovative approach to evaluate the impact of telepsychiatry on involuntary admissions, as well as the barriers and facilitators to implementing this method in the ED. Using telepsychiatry for ED assessment is currently not allowed as part of standard Israeli practice. With a waiver of the usual policy from the Ministry of Health, for the purpose of evaluation, we will implement telepsychiatry in 5 EDs throughout Israel. We will examine the effectiveness and implementation of telepsychiatry for involuntarily committed cases. We will compare administrative and clinical outcomes from the implementation phase with those from historical controls, as well as evaluate process outcomes using questionnaires. This study provides an opportunity to understand the feasibility of using this approach, as well as the impact of using this method on patients' outcomes. Our results will also help to provide policymakers with data to reassess the current policy.

This study is a response to the lack of evidence for telepsychiatry use in the ED. Other studies have examined telepsychiatry effectiveness in the outpatient setting [1,9]. There are some mixed studies from community and emergency settings examining telepsychiatry's effectiveness [40] or its implementation process [24]. To date, there are no studies that evaluate the use of telepsychiatry specifically for clinicians to evaluate the need for involuntary commitment. The issue of involuntary commitment involves some of the most vulnerable patients in the entire medical system, and there is a strong impetus to evaluate these patients promptly and help them reach a quieter and more stable environment more quickly. To the extent that telepsychiatry may help accomplish this goal, the study we present in this paper is crucially important to the telepsychiatry field.

This study has the potential to directly impact patient care and welfare as well as health systems. Our proposed study could improve patient safety and well-being by preventing long waits and, thus, opportunities for decompensation. We expect to show that this approach allows patients to be put in a safer place faster and spend less time in uncontrolled settings. We hypothesize that this will be expressed as fewer violent incidents and shorter hospital stays. There may also be an impact on ED crowding, which we will not directly measure. Patients may also receive direct therapeutic benefit. Telepsychiatry may be used to initiate psychiatric evaluation faster, reduce the length of the ED wait, and reduce decompensation. In turn, implementing telepsychiatry in the ED could reduce the costs and workload in the ED by moving patients out quicker. To the extent that we are able to show such an outcome, this will constitute a compelling argument for its widespread adoption.

### Limitations

This study has several potential challenges. There may be barriers to implementation, such as difficulty using video equipment or software, adequacy of Wi-Fi, or inability to reach the attending physician. There may also be resistance to change, as could occur with any change in practice. Since this is a “real world” study, another challenge is matching telepsychiatry to a variety of patients' backgrounds and contexts. Since motivation and intention are required on behalf of the patient in a video session [11,12], the ability of patients to fully cooperate may be an issue, especially since these are patients in the midst of a psychiatric emergency. By conducting this study, we will be able to identify such gaps and address them accordingly, or at least to quantify them.

### Conclusions

The implementation of telepsychiatry in the ED setting has the potential to improve care for patients. Although its efficacy has been noted in community settings, its application in emergency contexts remains largely uncharted territory. This study aims to shed light on the effectiveness of telepsychiatry for evaluations of involuntary commitment in the ED, as well as exploring the facilitators and barriers to its implementation in this setting. If our hypotheses are confirmed, it will not only demonstrate the accuracy of telepsychiatry but also pave the way for a more patient-centered and efficient approach in the psychiatric ED.

## Abbreviations

ED: emergency department
PARIHS: Promoting Action on Research Implementation in Health Services
SI: successful implementation

## Data Availability

The data sets that will be used and analyzed during the current study will be available from the corresponding author on reasonable request.
